# Locally Encoded Secure Distributed Batch Matrix Multiplication

**DOI:** 10.3390/e27121231

**Published:** 2025-12-05

**Authors:** Haobo Jia, Zhuqing Jia

**Affiliations:** School of Artificial Intelligence, Beijing University of Posts and Telecommunications, Beijing 100876, China; jiahaobo@bupt.edu.cn

**Keywords:** secure distributed computating, batch matrix multiplication, local encoding pattern, straggler tolerance, communication and computation efficiency

## Abstract

We study the problem of locally encoded secure distributed batch matrix multiplication (LESDBMM), where *M* pairs of sources each encode their respective batches of massive matrices and distribute the generated shares to a subset of *N* worker nodes. Each worker node computes a response from the received shares and sends the result to a sink node, which must be able to recover all *M* batches of pairwise matrix products in the presence of up to *S* stragglers. Additionally, any set of up to *X* colluding workers cannot learn any information about the matrices. Based on the idea of cross-subspace (CSA) codes and CSA null shaper, we propose the first LESDBMM scheme for batch processing. When the problem reduces to the coded distributed batch matrix multiplication (CDBMM) setting where M=1,X=0 and every source distributes its share to all worker nodes, the proposed scheme achieves performance matching that of the cross-subspace alignment (CSA) codes for CDBMM in terms of the maximum number of tolerable stragglers, communication cost, and computational complexity. Therefore, our scheme can be viewed as a generalization of CSA codes for CDBMM to the LESDBMM setting.

## 1. Introduction

The rapid advancement of big data processing and artificial intelligence technology has created substantial demand for low-latency computation of large-scale batch matrix multiplication, thereby motivating the emergence of the coded distributed batch matrix multiplication (CDBMM) problem. CDBMM aims to enable efficient and reliable distributed computation of a sequence of matrix products $AB=(A_{1}B_{1},A_{2}B_{2},\cdots ,A_{L}B_{L})$ by leveraging coding techniques to mitigate the impact of stragglers. Specifically, the sources encode two batches of matrices $A=(A_{1},A_{2},\cdots ,A_{L})$ and $B=(B_{1},B_{2},\cdots ,B_{L})$ into shares, which are then distributed to *N* worker nodes. Each worker then computes a response from received shares and sends it to a sink node. Some worker nodes may fail to send their response in time due to unforeseen factors such as network latency, hardware failures, or resource contention; these nodes are referred to as stragglers. The sink node must be able to recover the desired matrix multiplication results from the received responses, provided that the number of stragglers remains within a tolerable limit. Early approaches adopt Maximum Distance Separable (MDS) codes [1] and simply replicate half of the matrices unchanged across all worker nodes without encoding, leading to high computational overhead at workers. To reduce worker complexity, polynomial codes [2] introduced algebraic encoding via polynomial evaluation, achieving the optimal recovery threshold under a specific matrix partitioning strategy, namely, partitioning matrix $A_{l}$ row-wise and matrix $B_{l}$ column-wise for each l∈[L]. Subsequent works such as MatDot and PolyDot codes [3] further optimized the trade-off between communication load and recovery threshold by exploiting matrix partitioning along both rows and columns. Generalized PolyDot [4] improve the recovery threshold of PolyDot codes by a factor of 2, while Entangled Polynomial (EP) codes [5] generalize polynomial codes to support arbitrary matrix partitioning and achieve the same recovery threshold as Generalized PolyDot. Building on EP codes, the scheme in [6] designed a flexible coding strategy that dynamically adapts to the number of stragglers and optimizes download cost by utilizing all non-straggling workers. Lagrange Coded Computing (LCC) [7] extends these ideas to general batched computations using polynomial interpolation over finite fields, supporting both straggler resilience and privacy. Generalized Cross-Subspace Alignment (GCSA) codes [8] provides a powerful unifying framework that simultaneously supports arbitrary matrix partitioning and batch processing, achieving the best known performance. Meanwhile, the growing demand for privacy protection has motivated the study of secure distributed batch matrix multiplication (SDBMM). To prevent adversaries from learning information about the two batches of matrices A and B from the shares distributed to a subset of worker nodes, SDBMM requires any *X* colluding worker to not learn any information about the input matrices. Early secure extensions adapt polynomial codes to the SDBMM setting [9,10]. GASP codes [11] optimize download cost by tailoring the degrees of encoding polynomials to the partition parameters and the security threshold *X*. The authors of [12] propose two SDBMM schemes: one based on structured secret sharing and another built upon CSA codes. Generalized PolyDot codes are extended to SDBMM in [13], offering a tunable trade-off between recovery threshold and communication cost, and this framework is further generalized in [14] to support arbitrary collusion patterns. Bivariate polynomial codes are adapted to SDBMM in [15], balancing upload cost against average worker computation time. More recently, algebraic geometry codes have been employed to construct SDBMM schemes [16]. For scenarios requiring source privacy, where even the sink must remain ignorant of the input matrices A and B, polynomial sharing [17] uses secret sharing among workers to protect the inputs. However, it requires each worker node to securely exchange intermediate products with all others, resulting in significant inter-worker communication overhead. GCSA-NA codes [18] address this issue by allowing workers to pre-share randomness that is independent of the input matrices, thereby reducing inter-worker communication by orders of magnitude. Finally, the scheme in [19] improves upon *X*-secure GCSA [8] by achieving a lower recovery threshold for certain values of the security parameter *X*.

While the aforementioned CDBMM and SDBMM frameworks have achieved remarkable progress in straggler mitigation and security guarantees, they commonly rely on a crucial yet often unrealistic assumption: that every source node can distribute encoded shares to all worker nodes. This full-connectivity assumption greatly simplifies code design but fails to reflect practical distributed computing environments, such as edge computing clusters, federated learning systems, or data centers with hierarchical network topologies, where communication links may be restricted due to bandwidth limitations, access control policies, or physical proximity constraints. It is precisely this gap between the idealized model of CDBMM and real-world deployment scenarios that motivates our work. When source–worker connectivity becomes partial, the conventional global encoding strategies (e.g., EP codes, LCC codes) are no longer directly applicable, as each source can only influence a subset of workers. This necessitates a new coding paradigm that respects the locality of encoding while preserving straggler tolerance and security, naturally leading to the formulation of the locally encoded secure distributed batch matrix multiplication (LESDBMM) problem. The LESDBMM problem involves *M* pairs of source nodes $\left(S_{A,1},S_{B,1}\right)$, $\left(S_{A,2},S_{B,2}\right)$, ⋯, $\left(S_{A,M},S_{B,M}\right)$, *N* worker nodes, and one sink node. The sink node can communicate with all worker nodes, while each pair of source nodes is connected only to a subset of worker nodes. The communication connectivity between all source nodes and worker nodes is globally known. Each pair of source nodes $S_{A,m}$ and $S_{B,m}$ encodes their batches of matrices $A_{m}=\left(A_{m,1},A_{m,2},\cdots ,A_{m,L}\right)$ and $B_{m}=\left(B_{m,1},B_{m,2},\cdots ,B_{m,L}\right)$ and distributes the generated shares exclusively to its connected worker nodes; such an encoding pattern is referred to as “local encoding pattern”. The worker nodes must remain oblivious to the values of the matrices ${\left(A_{m},B_{m}\right)}_{m\in [M]}$. Any set of up to *X* colluding workers must learn no information about these matrices. Each worker node computes a response from the received shares and sends it to the sink node. The sink node must be able to recover the desired matrix multiplication ${\left(A_{m,1}B_{m,1},A_{m,2}B_{m,2},\cdots ,A_{m,L}B_{m,L}\right)}_{m\in [M]}$ as long as the number of stragglers does not exceed *S*. This work aims to construct an efficient and straggler-tolerant LESDBMM scheme. The key challenge lies in leveraging the local encoding pattern to design an encoding scheme, such that the interference across different encoding subsets can be aligned into as few dimensions as possible, thereby minimizing the communication cost required for interference elimination during decoding. Furthermore, the decoding scheme must mitigate the impact of randomly occurring stragglers on the locally encoded structure.

A closely related problem is the problem of *X*-secure *T*-private linear computation based on graph-based replicated/MDS-coded storage (GXSTPLC) [20]. In the GXSTPLC problem, *K* messages are partitioned into *M* message sets, and the messages of each message set are restricted to be distributed among a subset of *N* servers in a securely coded form. Any set of *X* colluding servers must not disclose any information about the stored messages. A user wishes to privately compute a linear combination of all messages. To this end, the user sends queries to the servers and recovers the desired linear combination from the answer returned by servers. In this process, all servers must remain available, and any *T* colluding server must learn nothing about the coefficients of the linear combination. Ref. [20] proposes the first asymptotic capacity achieving the GXSTPLC scheme for replicated storage based on the idea of cross-subspace alignment (CSA) and a structure inspired by dual generalized Reed–Solomon (GRS) codes, demonstrating the optimality of CSA codes and dual GRS codes for interference alignment across message sets in this setting. Ref. [21] proposes a GXSTPLC scheme for MDS-coded storage based on CSA codes and exploits the idea of CSA null shaper, rather than using dual GRS codes, to enable interference alignment across message sets. When applied to the case of replicated storage, its rate matches the asymptotic capacity established in [20]. In fact, there is a connection between the LESDBMM problem and the GXSTPLC problem. If we let $A_{m,l}$ be a row vector and $B_{m,l}$ be a column vector, the desired matrix multiplication ${\left(A_{m,1}B_{m,1},A_{m,2}B_{m,2},\cdots ,A_{m,L}B_{m,L}\right)}_{m\in [M]}$ then degenerates into *M* batches of pairwise vector inner products. By thinking of $A_{m,l}$ as the $l^{th}$ symbols of each message in the $m^{th}$ message set and $B_{m,l}$ as corresponding coefficients, any GXSTPLC scheme automatically yields an LESDBMM scheme. This work focuses on extending the batch vector inner product scheme yielded by the MDS-GXSTPLC scheme [21] to an LESDBMM scheme applicable to matrix multiplication of arbitrary dimensions and enabling it to tolerate stragglers.

The main contribution of this work is the first LESDBMM scheme based on batch processing. Our scheme utilizes CSA codes and CSA null shaper to achieve interference alignment across encoding subsets. We evaluate the scheme in terms of the straggler threshold (i.e., maximum number of tolerable stragglers), upload cost, download cost, encoding complexity, worker node computation complexity, and decoding complexity. By comparing with the baseline scheme, we demonstrate that the optimization of download cost achieved by our scheme is non-trivial. Moreover, by adjusting the parameter $K_{c}$, we can achieve a trade-off between performance metrics of the encoding and decoding phases. When the problem degenerates to the CDBMM setting, where M=1,X=0 and all source nodes can distribute their shares to all workers, the performance of our scheme matches that of the CSA codes for CDBMM [8]. Hence, our scheme can be viewed as a generalization of the CSA codes for CDBMM to the LESDBMM setting.

The remainder of this paper is organized as follows. Section 2 formally defines the problem of locally encoded secure distributed batch matrix multiplication. Section 3 presents the main result. Section 4 presents the proof of our main result along with an illustrative example. Section 5 concludes the paper and discusses future research directions.

*Notation:* Bold symbols are used to denote vectors and matrices, while calligraphic symbols denote sets. Following the convention, let the empty product be the multiplicative identity and the empty sum be the additive identity. For any two positive integers m<n, [m:n] denotes the set {m,m+1,⋯,n}. We use the shorthand notation [n] for [1:n]. For an index set $I=\{i_{1},i_{2},\cdots ,i_{m}\}$, $X_{I}$ denotes the set $\left\{X_{i_{1}},X_{i_{2}},\cdots ,X_{i_{m}}\right\}$. For a subset of integers C, C(i),i∈[|C|] denotes its $i^{th}$ element in ascending order. For an m×n matrix V and two integers a∈[m],b∈[n], V(a,b) denotes the element at the $a^{th}$ row and the $b^{th}$ column of V. $0_{m\times n}$ denotes the zero matrix of size m×n. The notation $\tilde{O}$ suppresses polylog terms; i.e., $\tilde{O}\left(n\log^{2}m\right)$ can be replaced with $O(n\log^{2}m)$ if the field F supports the Fast Fourier Transform (FFT) and with $O(n\log^{2}m\log\log\left(m\right))$ if it does not.

## 2. Problem Statement

**Figure 1:**
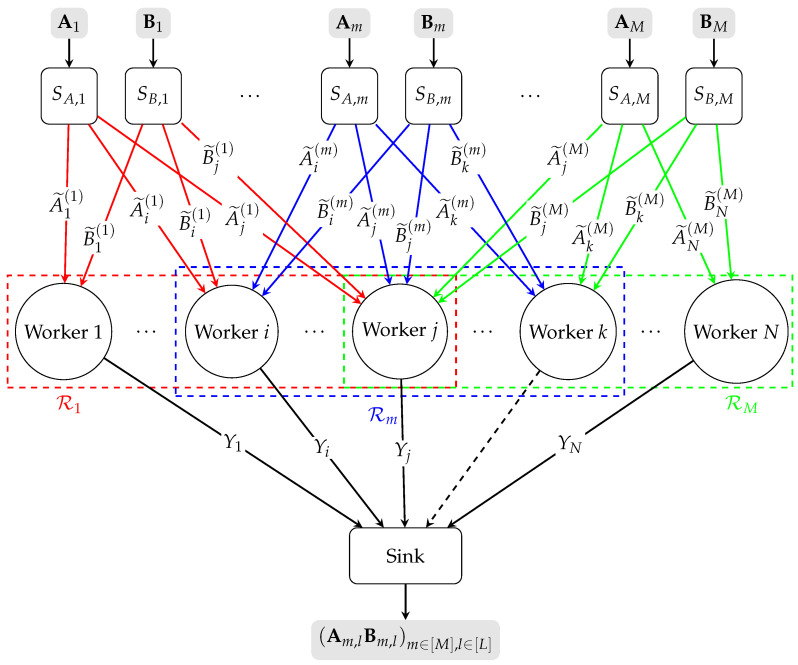
Conceptual diagram of the LESDBMM problem with *M* pairs of source nodes, *N* worker nodes, and local encoding pattern $\left\{R_{1},\cdots ,R_{m},\cdots ,R_{M}\right\}$. The worker node *k* is a straggler. The ellipsis between two nodes indicates that the intermediate nodes between them have been omitted. Lines in different colors represent distinct local connectivity patterns between different sources and the workers. Dashed lines connecting a worker to the sink indicate that the worker is straggling and cannot return any information to the sink.

Consider the LESDBMM problem as shown in Figure 1 with *M* pairs of source nodes $\left(S_{A,1},S_{B,1}\right)$, $\left(S_{A,2},S_{B,2}\right)$, ⋯, $\left(S_{A,M},S_{B,M}\right)$ and *N* worker nodes 1,2,⋯,N. Each source node $S_{A,m}$, m∈[M] generates a batch of matrices $A_{m}=\left(A_{m,1},A_{m,2},\cdots ,A_{m,L}\right)$, where for all l∈[L], $A_{m,l}\in F_{q}^{\lambda \times \kappa }$. Each source node $S_{B,m}$, m∈[M] generates a batch of matrices $B_{m}=\left(B_{m,1},B_{m,2},\cdots ,B_{m,L}\right)$, where for all l∈[L], $B_{m,l}\in F_{q}^{\kappa \times \mu }$. A sink node with limited computing power demands ${\left(A_{m,l}B_{m,l}\right)}_{m\in [M],l\in [L]}$. For this purpose, the sink node requires each source node to encode and send its matrices to *N* worker nodes and have them assist in computing matrix multiplications. Due to constrained communication links between the source nodes and the worker nodes, each pair of source nodes $S_{A,m},S_{B,m},\;m\in \left[M\right]$ can only send their encoded matrices to a subset of *N* worker nodes denoted as $R_{m}=\left\{R_{m}\left(1\right),R_{m}\left(2\right),\cdots ,R_{m}\left(\rho_{m}\right)\right\}$, $0<\rho_{m}\leq N$, i.e., $R_{m}\subseteq \left[N\right]$. The collection of sets $\left\{R_{m}|m\in \left[M\right]\right\}$ is referred to as “local encoding pattern”. We can equivalently define the dual representation of the local encoding pattern. For all n∈[N], let us define the index set of encoded batches of matrices that are available at worker node *n* as $M_{n}=\left\{m\in \left[M\right]|n\in R_{m}\right\}$.

Each source node $S_{A,m},\;m\in \left[M\right]$ encodes the matrices $A_{m}$ according to functions ${\left(f_{n}^{\left(m\right)}\right)}_{n\in R_{m}}$ and generates the shares ${\left({\tilde{A}}_{n}^{\left(m\right)}\right)}_{n\in R_{m}}$, where for all $n\in R_{m}$, ${\tilde{A}}_{n}^{\left(m\right)}=f_{n}^{\left(m\right)}\left(A_{m}\right)$ is sent to worker node *n*. Each source node $S_{B,m},\;m\in \left[M\right]$ encodes the matrices $B_{m}$ according to functions ${\left(g_{n}^{\left(m\right)}\right)}_{n\in R_{m}}$ and generates the shares ${\left({\tilde{B}}_{n}^{\left(m\right)}\right)}_{n\in R_{m}}$, where for all $n\in R_{m}$, ${\tilde{B}}_{n}^{\left(m\right)}=g_{n}^{\left(m\right)}\left(B_{m}\right)$ is sent to worker node *n*. The shares of *M* batches of matrices ${\left(A_{m},B_{m}\right)}_{m\in [M]}$ are generated independently, i.e.,

(1) $$\begin{array}{c} H\left({\left({\tilde{A}}_{n}^{\left(m\right)},{\tilde{B}}_{n}^{\left(m\right)}\right)}_{m\in \left[M\right],n\in R_{m}}\right)=\sum_{m\in [M]}H\left({\left({\tilde{A}}_{n}^{\left(m\right)}\right)}_{n\in R_{m}}\right)+\sum_{m\in [M]}H\left({\left({\tilde{B}}_{n}^{\left(m\right)}\right)}_{n\in R_{m}}\right). \end{array}$$

Any group of up to *X* colluding worker nodes cannot learn any information about the matrices ${\left(A_{m},B_{m}\right)}_{m\in [M]}$; i.e., for all m∈[M],

(2) $$\begin{array}{c} I\left(A_{m},B_{m};{\left({\tilde{A}}_{n}^{\left(m\right)},{\tilde{B}}_{n}^{\left(m\right)}\right)}_{n\in X}\right)=0,\;\forall X\subset R_{m},\;\left|X\right|=X. \end{array}$$

Upon receiving shares ${\left({\tilde{A}}_{n}^{\left(m\right)},{\tilde{B}}_{n}^{\left(m\right)}\right)}_{m\in M_{n}}$, each worker node n,n∈[N] computes a response $Y_{n}$ according to function $h_{n}$, i.e.,

(3) $$\begin{array}{c} Y_{n}=h_{n}\left({\left({\tilde{A}}_{n}^{\left(m\right)},{\tilde{B}}_{n}^{\left(m\right)}\right)}_{m\in M_{n}}\right), \end{array}$$

and sends $Y_{n}$ to the sink node.

The sink node must be able to decode the desired products ${\left(A_{m,l}B_{m,l}\right)}_{m\in [M],l\in [L]}$ in the presence of up to *S* stragglers. Let T denote the index set of the N−S fastest worker nodes that send responses to the sink node. For any subset T⊂[N], according to decoding function $d_{T}$, we have

(4) $$\begin{array}{c} {\left(A_{m,l}B_{m,l}\right)}_{m\in [M],l\in [L]}=d_{T}\left(Y_{T}\right). \end{array}$$

Let us denote $f={\left(f_{n}^{\left(m\right)}\right)}_{m\in \left[M\right],n\in R_{m}},g={\left(g_{n}^{\left(m\right)}\right)}_{m\in \left[M\right],n\in R_{m}},h=h_{\left[N\right]},d={\left(d_{T}\right)}_{T\subset [N]}$. We say that (f,g,h,d) form an LESDBMM scheme against *S* stragglers. To evaluate the performance of an LESDBMM scheme defined above, we consider the straggler threshold, the communication cost, and computation complexity. The straggler threshold *S* is defined as the maximum number of tolerable stragglers. The communication cost includes upload cost $\left(U_{A},U_{B}\right)$ and download cost *D*, defined as follows:

(5) $$\begin{array}{cc} \; & U_{A}=\frac{\sum_{m\in [M]}\sum_{n\in R_{m}}\left|{\tilde{A}}_{n}^{\left(m\right)}\right|}{ML\lambda \kappa }, \end{array}$$

(6) $$\begin{array}{cc} \; & U_{B}=\frac{\sum_{m\in [M]}\sum_{n\in R_{m}}\left|{\tilde{B}}_{n}^{\left(m\right)}\right|}{ML\kappa \mu }, \end{array}$$

(7) $$\begin{array}{cc} \; & D=\frac{\sum_{n\in T}\left|Y_{n}\right|}{ML\lambda \mu }, \end{array}$$

where |C| counts the number of symbols from $F_{q}$ needed to represent *C*. The computation complexity includes encoding complexity $\left(C_{eA},C_{eB}\right)$, worker node computation complexity $C_{w}$, and decoding complexity $C_{d}$. $C_{eA}$ and $C_{eB}$ are defined as the order of the number of finite field arithmetic operations required to compute f and g, normalized by ML. $C_{w}$ is defined as the order of the number of finite field arithmetic operations required to compute h, normalized by ML. $C_{d}$ is defined as the order of the number of finite field arithmetic operations required to compute $d_{T}\left(Y_{T}\right)$, normalized by ML.

## 3. Main Result

Consider a hypergraph G=(V,E) with vertex set V=[N] and hyperedge set $E=\{R_{m}|m\in \left[M\right]\}$, where each vertex represents a worker node and each hyperedge indicates that the vertices it contains share a common piece of information. Suppose that G consists of *K* connected components, denoted by $G_{k}=\left(V_{k},E_{k}\right)$ for k=1,2,…,K. For all k∈[K], let $I_{k}$ denote the index set of the hyperedges contained in $E_{k}$, i.e., $E_{k}=\left\{R_{m}|m\in I_{k}\right\}$.

The main result of this work is stated in the following theorem.

**Theorem** **1.** *For LESDBMM over the finite field $F_{q}$ with N worker nodes, local encoding pattern $\left\{R_{m}|m\in \left[M\right]\right\}$, positive integers $\ell ,K_{c}$ such that $L=\ell K_{c}\leq q-N$ and $\min_{k\in [K]}(\rho_{\min}^{\left(k\right)}-|I_{k}\left|L\right)>2X+K_{c}-1$, the following straggler threshold, communication cost, and computation complexity can be achieved:*

(8) $$\begin{array}{c} \mathrm{Straggler}\mathrm{Threshold}:\;S=\min_{k\in [K]}\rho_{\min}^{\left(k\right)}-\left|I_{k}\right|L-2X-K_{c}+1, \end{array}$$

(9) $$\begin{array}{c} \mathrm{Upload}\mathrm{Cost}:\;\left(U_{A},U_{B}\right)=\left(\frac{\sum_{m\in [M]}\rho_{m}}{MK_{c}},\frac{\sum_{m\in [M]}\rho_{m}}{MK_{c}}\right), \end{array}$$

(10) $$\begin{array}{c} \mathrm{Dowload}\mathrm{Cost}:\;D=\frac{\sum_{k\in [K]}\gamma_{k}}{ML}, \end{array}$$

(11) $$\begin{array}{c} \;\;\mathrm{Encoding}\mathrm{Complexity}:\\ \;\left(C_{eA},C_{eB}\right)=\left(\tilde{O}\left(\frac{\lambda \kappa \sum_{m\in [M]}\rho_{m}\log^{2}\rho_{m}}{MK_{c}}\right),\tilde{O}\left(\frac{\kappa \mu \sum_{m\in [M]}\rho_{m}\log^{2}\rho_{m}}{MK_{c}}\right)\right), \end{array}$$

(12) $$\begin{array}{c} \mathrm{Worker}\mathrm{Node}\mathrm{Computation}\mathrm{Complexity}:\;C_{w}=O\left(\frac{\lambda \kappa \mu \sum_{m\in [M]}\rho_{m}}{MK_{c}}\right) \end{array}$$

(13) $$\begin{array}{c} \mathrm{Decoding}\mathrm{Complexity}:\;C_{d}=\tilde{O}\left(\frac{\lambda \mu \sum_{k\in [K]}\gamma_{k}\log^{2}\gamma_{k}}{ML}\right), \end{array}$$

*where for all k∈[K], $\rho_{\min}^{\left(k\right)}≜\min_{m\in I_{k}}\rho_{m},\gamma_{k}≜|V_{k}|-(\rho_{\min}^{\left(k\right)}-|I_{k}\left|L-2X-K_{c}+1\right)$.*

**Remark** **1.** *If hypergraph $G=(\left[N\right],\left\{R_{m}|m\in \left[M\right]\right\})$ is connected, we have the straggler threshold $S=\min_{m\in [M]}\rho_{m}-2X-K_{c}-ML+1$ and the decoding complexity $C_{d}=\tilde{O}\left(\lambda \mu \left(N-S\right)\log^{2}\left(N-S\right)/\left(ML\right)\right)=\tilde{O}\left(\lambda \mu D\log^{2}\left(N-S\right)\right)$. It can be seen that the straggler threshold is determined by the minimum number of worker nodes that any source node can communicate with. When the problem degenerates to the CDBMM setting; i.e., when $M=1,R_{1}=\left[N\right]$ and X=0, the straggler threshold, upload cost, download cost, encoding complexity, computation complexity of all worker nodes, and decoding complexity achieved by Theorem 1 match those of CSA codes for CDBMM [8].*

**Remark** **2.** *We say that two pairs of source nodes $\left(S_{A,m},S_{B,m}\right)$ and $\left(S_{A,m^{\prime }},S_{B,m^{\prime }}\right)$ are related if the sets of worker nodes connected to them belong to the same connected component; i.e., there exists k∈[K], such that $R_{m}\subset V_{k},R_{m^{\prime }}\subset V_{k}$. When the connectivity between a given pair of source nodes $\left(S_{A,m},S_{B,m}\right)$ and the worker nodes is too sparse (i.e., $\rho_{m}$ is too small) and the number of pairs of source nodes related to $\left(S_{A,m},S_{B,m}\right)$ is excessively large, condition $\min_{k\in [K]}(\rho_{\min}^{\left(k\right)}-|I_{k}\left|L\right)>2X+K_{c}-1$ cannot be satisfied even when we set $K_{c}=1$, rendering Theorem 1 inapplicable. A trivial approach to resolving this problem is to exclude the source node pairs possessing this property from the original local encoding pattern and handle the computational tasks of each of these pairs of source nodes separately. However, this scheme evidently incurs significant communication and computational overhead.*

**Remark** **3.** *The parameter $K_{c}$ directly governs key performance trade-offs: increasing $K_{c}$ reduces upload cost, encoding complexity at the source nodes, and worker node computation complexity, but it decreases the straggler threshold and increases download cost and decoding complexity at the sink node.*

**Remark** **4.** *Compared to the baseline scheme shown in Table 1, our scheme achieves a lower download cost under the same $K_{c}$, at the expense of a potentially reduced straggler threshold. However, because the baseline scheme lacks interference alignment across encoding subsets, its download cost remains higher than that of our scheme, even when its parameter $K_{c}$ is increased to reduce the straggler threshold to match ours. For instance, in the motivating example described in Section 4.1 with a straggler threshold of 1 and worker node 2 failing, our scheme achieves a download cost of 19/12, whereas the baseline requires 7/4.*

## 4. The Proof of Theorem 1

### 4.1. Motivating Example

**Figure 2:**
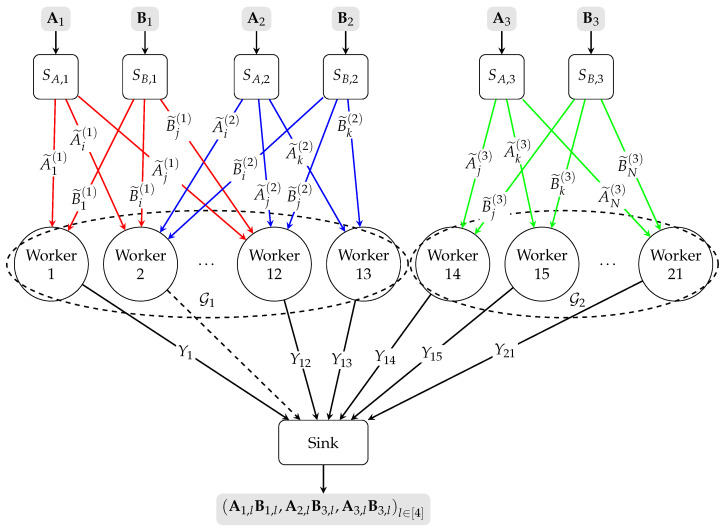
Conceptual diagram of the LESDBMM problem with M=3 pairs of source nodes, N=21 worker nodes, and local encoding pattern $\left\{R_{1}=\left\{\left[12\right]\right\},R_{2}=\left\{\left[2:13\right]\right\},R_{3}=\left\{\left[14:21\right]\right\}\right\}$. The hypergraph $G=(\left[N\right],\left\{R_{1},R_{2},R_{3}\right\})$ has two connected components $G_{1}=\left(\left[13\right],\left\{\left[12\right],\left[2:13\right]\right\}\right)$ and $G_{2}=\left(\left[14:21\right],\left\{\left[14:21\right]\right\}\right)$. The worker node 2 is a straggler. The ellipsis between two nodes indicates that the intermediate nodes between them have been omitted. Lines in different colors represent distinct local connectivity patterns between different sources and the workers. Dashed lines connecting a worker to the sink indicate that the worker is straggling and cannot return any information to the sink.

To make the general proof more accessible, let us consider a motivating example as shown in Figure 2 where we have M=3 pairs of source nodes $\left(S_{A,1},S_{B,1}\right)$, $\left(S_{A,2},S_{B,2}\right)$, and $\left(S_{A,3},S_{B,3}\right)$, N=21 worker nodes, batch size L=4, and security level X=1. The local encoding pattern for this example is $R_{1}=\left[12\right],\rho_{1}=12,R_{2}=\left[2:13\right],\rho_{2}=12,R_{3}=\left[14:21\right],\rho_{3}=8$. Accordingly, we have $M_{1}=\left\{1\right\}$, $M_{n}=\left\{1,2\right\}$ for all n∈[2:12], $M_{13}=\left\{2\right\}$, and $M_{n}=\left\{3\right\}$ for all n∈[14:21]. For all m∈[3], source node $S_{A,m}$ generates a batch of matrices as follows:

(14) $$\begin{array}{c} A_{m}=\left(A_{m,1},A_{m,2},A_{m,3},A_{m,4}\right), \end{array}$$

Source node $S_{B,m}$ generates a batch of matrices as follows:

(15) $$\begin{array}{c} B_{m}=\left(B_{m,1},B_{m,2},B_{m,3},B_{m,4}\right). \end{array}$$

The hypergraph G=([13],{[12],[2:13],[14:21]}) has two connected components $G_{1}=\left(\left[13\right],\left\{\left[12\right],\left[2:13\right]\right\}\right)$ and $G_{2}=\left(\left[14:21\right],\left\{\left[14:21\right]\right\}\right)$, thus $\rho_{\min}^{\left(1\right)}=12,\rho_{\min}^{\left(2\right)}=8$. Let us set $\ell =2,K_{c}=2$, then we have $S=\min\left(\rho_{\min}^{\left(1\right)}-2L,\rho_{\min}^{\left(2\right)}-L\right)-2X-K_{c}+1=1$; i.e., the scheme can tolerate one straggler.

Let $\alpha_{1},\alpha_{2},\cdots ,\alpha_{21},{\left(f_{m,1,1},f_{m,1,2},f_{m,2,1},f_{m,2,2}\right)}_{m\in [3]}$ be 33 distinct elements from $F_{q}$, thus we need q≥33. For all m∈[3], let us define

(16) $$\begin{array}{cccc} A_{m,1,1}=A_{m,1},\;\;\;\;\; & A_{m,1,2}=A_{m,2},\;\;\;\;\; & A_{m,2,1}=A_{m,3},\;\;\;\;\; & A_{m,2,2}=A_{m,4}, \end{array}$$

(17) $$\begin{array}{cccc} B_{m,1,1}=B_{m,1},\;\;\;\;\; & B_{m,1,2}=B_{m,2},\;\;\;\;\; & B_{m,2,1}=B_{m,3},\;\;\;\;\; & B_{m,2,2}=B_{m,4}. \end{array}$$

#### 4.1.1. Encoding

For all $n\in \left[21\right],m\in M_{n},l\in \left[2\right]$, let us define the following constants:

(18) $$\begin{array}{c} \Delta_{n,m,l}=\left(\alpha_{n}-f_{m,l,1}\right)\left(\alpha_{n}-f_{m,l,2}\right). \end{array}$$

Let ${\left(Z_{m,1}^{\left(A\right)},Z_{m,2}^{\left(A\right)}\right)}_{m\in [3]},$ be six uniformly i.i.d. random matrices over $F_{q}^{\lambda \times \kappa }$; let ${\left(Z_{m,1}^{\left(B\right)},Z_{m,2}^{\left(B\right)}\right)}_{m\in [3]}$ be six uniformly i.i.d. random matrices over $F_{q}^{\kappa \times \mu }$.

For all $m\in \left[3\right],n\in R_{m}$, the share uploaded to worker node *n* by source node $S_{A,m}$ is constructed as follows:

(19) $$\begin{array}{cc} {\tilde{A}}_{n}^{\left(m\right)} & =\left({\bar{A}}_{n,1}^{\left(m\right)},{\bar{A}}_{n,2}^{\left(m\right)}\right), \end{array}$$

where

(20) $$\begin{array}{cc} {\bar{A}}_{n,1}^{\left(m\right)}= & \Delta_{n,m,1}\left(\frac{1}{\alpha_{n}-f_{m,1,1}}A_{m,1,1}+\frac{1}{\alpha_{n}-f_{m,1,2}}A_{m,1,2}+Z_{m,1}^{\left(A\right)}\right), \end{array}$$

(21) $$\begin{array}{ccc} & = & \left(\alpha_{n}-f_{m,1,2}\right)A_{m,1,1}+\left(\alpha_{n}-f_{m,1,1}\right)A_{m,1,2}+\Delta_{n,m,1}Z_{m,1}^{\left(A\right)} \end{array}$$

(22) $$\begin{array}{cc} {\bar{A}}_{n,2}^{\left(m\right)}= & \Delta_{n,m,2}\left(\frac{1}{\alpha_{n}-f_{m,2,1}}A_{m,2,1}+\frac{1}{\alpha_{n}-f_{m,2,2}}A_{m,2,2}+Z_{m,2}^{\left(A\right)}\right), \end{array}$$

(23) $$\begin{array}{ccc} & = & \left(\alpha_{n}-f_{m,2,2}\right)A_{m,2,1}+\left(\alpha_{n}-f_{m,2,1}\right)A_{m,2,2}+\Delta_{n,m,2}Z_{m,2}^{\left(A\right)} \end{array}$$

The share uploaded to worker node *n* by source node $S_{B,m}$ is constructed as follows:

(24) $$\begin{array}{cc} {\tilde{B}}_{n}^{\left(m\right)} & =\left({\bar{B}}_{n,1}^{\left(m\right)},{\bar{B}}_{n,2}^{\left(m\right)}\right), \end{array}$$

where

(25) $$\begin{array}{cc} \; & {\bar{B}}_{n,1}^{\left(m\right)}=\frac{1}{\alpha_{n}-f_{m,1,1}}B_{m,1,1}+\frac{1}{\alpha_{n}-f_{m,1,2}}B_{m,1,2}+Z_{m,1}^{\left(B\right)}, \end{array}$$

(26) $$\begin{array}{cc} \; & {\bar{B}}_{n,2}^{\left(m\right)}=\frac{1}{\alpha_{n}-f_{m,2,1}}B_{m,2,1}+\frac{1}{\alpha_{n}-f_{m,2,2}}B_{m,2,2}+Z_{m,2}^{\left(B\right)}. \end{array}$$

Note that any single worker node cannot reveal any information about the matrices $A_{m,1,1},A_{m,1,2}A_{m,2,1},A_{m,2,2}$ and $B_{m,1,1},B_{m,1,2}B_{m,2,1},B_{m,2,2}$ due to the random noise matrices $Z_{m,1}^{\left(A\right)},Z_{m,2}^{\left(A\right)},Z_{m,1}^{\left(B\right)},Z_{m,2}^{\left(B\right)}$.

The upload cost is

(27) $$\begin{array}{cc} \; & U_{A}=\frac{2\lambda \kappa \sum_{m\in [3]}\rho_{m}}{ML\lambda \kappa }=\frac{16}{3}, \end{array}$$

(28) $$\begin{array}{cc} \; & U_{B}=\frac{2\kappa \mu \sum_{m\in [3]}\rho_{m}}{ML\kappa \mu }=\frac{16}{3}. \end{array}$$

The encoding complexity is

(29) $$\begin{array}{cc} \; & C_{eA}=O\left(\lambda \kappa \right) \end{array}$$

(30) $$\begin{array}{cc} \; & C_{eB}=O\left(\kappa \mu \right). \end{array}$$

Note that under the settings of this example, the straggler threshold for the baseline scheme is 1. The uploaded shares to worker node *n* by source nodes $S_{A,m}$ and $S_{B,m}$ in the baseline scheme are the same as those in (19) and (24), respectively, making the upload cost of the baseline scheme identical to that given in (27) and (24). Additionally, the encoding complexity of the baseline scheme matches that provided in (29) and (30).

#### 4.1.2. Computing

For all n∈[13], let us define $\Gamma_{1,n,1}=\left(\alpha_{n}-\alpha_{13}\right),\Gamma_{1,n,2}=\left(\alpha_{n}-\alpha_{1}\right)$. For all n∈[14:21], let us define $\Gamma_{2,n,3}=1$.

The response $Y_{1}$ is constructed as

(31) $$\begin{array}{cc} Y_{1}=Y_{1}= & \Gamma_{1,1,1}\left({\bar{A}}_{1,1}^{\left(1\right)}{\bar{B}}_{1,1}^{\left(1\right)}+{\bar{A}}_{1,2}^{\left(1\right)}{\bar{B}}_{1,2}^{\left(1\right)}\right) \end{array}$$

(32) $$\begin{array}{cc} = & \Gamma_{1,1,1}\left({\bar{A}}_{1,1}^{\left(1\right)}{\bar{B}}_{1,1}^{\left(1\right)}+{\bar{A}}_{1,2}^{\left(1\right)}{\bar{B}}_{1,2}^{\left(1\right)}\right)+0\times \left({\bar{A}}_{1,1}^{\left(2\right)}{\bar{B}}_{1,1}^{\left(2\right)}+{\bar{A}}_{1,2}^{\left(2\right)}{\bar{B}}_{1,2}^{\left(2\right)}\right) \end{array}$$

(33) $$\begin{array}{cc} = & \sum_{m\in [2]}\Gamma_{1,1,m}\left({\bar{A}}_{1,1}^{\left(m\right)}{\bar{B}}_{1,1}^{\left(m\right)}+{\bar{A}}_{1,2}^{\left(m\right)}{\bar{B}}_{1,2}^{\left(m\right)}\right) \end{array}$$

For all n∈[2:12], the response $Y_{n}$ is constructed as

(34) $$\begin{array}{cc} Y_{n}= & Y_{n}=\sum_{m\in [2]}\Gamma_{1,n,m}\left({\bar{A}}_{n,1}^{\left(m\right)}{\bar{B}}_{n,1}^{\left(m\right)}+{\bar{A}}_{n,2}^{\left(m\right)}{\bar{B}}_{n,2}^{\left(m\right)}\right). \end{array}$$

The response $Y_{13}$ is constructed as

(35) $$\begin{array}{cc} Y_{13}=Y_{13}= & \Gamma_{1,13,2}\left({\bar{A}}_{13,1}^{\left(2\right)}{\bar{B}}_{13,1}^{\left(2\right)}+{\bar{A}}_{13,2}^{\left(2\right)}{\bar{B}}_{13,2}^{\left(2\right)}\right) \end{array}$$

(36) $$\begin{array}{cc} = & 0\times \left({\bar{A}}_{13,1}^{\left(1\right)}{\bar{B}}_{13,1}^{\left(1\right)}+{\bar{A}}_{13,2}^{\left(1\right)}{\bar{B}}_{13,2}^{\left(1\right)}\right)+\Gamma_{1,13,2}\left({\bar{A}}_{13,1}^{\left(2\right)}{\bar{B}}_{13,1}^{\left(2\right)}+{\bar{A}}_{13,2}^{\left(2\right)}{\bar{B}}_{13,2}^{\left(2\right)}\right) \end{array}$$

(37) $$\begin{array}{cc} = & \sum_{m\in [2]}\Gamma_{1,13,m}\left({\bar{A}}_{13,1}^{\left(m\right)}{\bar{B}}_{13,1}^{\left(m\right)}+{\bar{A}}_{13,2}^{\left(m\right)}{\bar{B}}_{13,2}^{\left(m\right)}\right). \end{array}$$

Therefore, for all n∈[13], the response $Y_{n}$ can be formally written as

(38) $$\begin{array}{cc} Y_{n}= & Y_{n}=\sum_{m\in [2]}\Gamma_{1,n,m}\left({\bar{A}}_{n,1}^{\left(m\right)}{\bar{B}}_{n,1}^{\left(m\right)}+{\bar{A}}_{n,2}^{\left(m\right)}{\bar{B}}_{n,2}^{\left(m\right)}\right). \end{array}$$

For all n∈[14:21], the response $Y_{n}$ is constructed as

(39) $$\begin{array}{cc} Y_{n}=Y_{n}= & \Gamma_{2,n,3}\left({\bar{A}}_{n,1}^{\left(3\right)}{\bar{B}}_{n,1}^{\left(3\right)}+{\bar{A}}_{n,2}^{\left(3\right)}{\bar{B}}_{n,2}^{\left(3\right)}\right) \end{array}$$

(40) $$\begin{array}{cc} = & {\bar{A}}_{n,1}^{\left(3\right)}{\bar{B}}_{n,1}^{\left(3\right)}+{\bar{A}}_{n,2}^{\left(3\right)}{\bar{B}}_{n,2}^{\left(3\right)}. \end{array}$$

Specifically, for all $m\in \left[3\right],n\in R_{m}$, we obtain

(41) $$\begin{array}{cc} \; & \sum_{l\in [2]}{\bar{A}}_{n,l}^{\left(m\right)}{\bar{B}}_{n,l}^{\left(m\right)}\\ = & \sum_{l\in [2]}\left(\frac{f_{m,l,2}-f_{m,l,1}}{\alpha_{n}-f_{m,l,1}}A_{m,l,1}B_{m,l,1}+\frac{f_{m,l,1}-f_{m,l,2}}{\alpha_{n}-f_{m,l,2}}A_{m,l,2}B_{m,l,2}\right)+\sum_{z\in [3]}\alpha_{n}^{z-1}{\bar{Z}}_{m,z}, \end{array}$$

where for all m∈[2],z∈[3], ${\bar{Z}}_{m,z}$ are obtained by combining the like terms. For all $m\in \left[2\right],n\in R_{m}$, we obtain

(42) $$\begin{array}{cc} \; & \Gamma_{1,n,m}\sum_{l\in [2]}{\bar{A}}_{n,l}^{\left(m\right)}{\bar{B}}_{n,l}^{\left(m\right)}\\ = & \sum_{l\in [2]}\left(\frac{{\bar{c}}_{m,l,1}}{\alpha_{n}-f_{m,l,1}}A_{m,l,1}B_{m,l,1}+\frac{{\bar{c}}_{m,l,2}}{\alpha_{n}-f_{m,l,2}}A_{m,l,2}B_{m,l,2}\right)+\sum_{z\in [4]}\alpha_{n}^{z-1}{\tilde{Z}}_{m,z} \end{array}$$

where $\beta_{1}=\alpha_{13}$, $\beta_{2}=\alpha_{1}$, for all m∈[2],l∈[2],z∈[4], ${\bar{c}}_{m,l,1}=\left(f_{m,l,1}-\beta_{m}\right)\left(f_{m,l,2}-f_{m,l,1}\right),{\bar{c}}_{m,l,2}=\left(f_{m,l,1}-\beta_{m}\right)\left(f_{m,l,1}-f_{m,l,2}\right)$${\tilde{Z}}_{m,z}$ are obtained by combining the like terms.

Therefore, for all n∈[13], $Y_{n}$ can be written as

(43) $$\begin{array}{cc} Y_{n}= & \sum_{m\in [2]}\Gamma_{1,n,m}\left({\bar{A}}_{n,1}^{\left(m\right)}{\bar{B}}_{n,1}^{\left(m\right)}+{\bar{A}}_{n,2}^{\left(m\right)}{\bar{B}}_{n,2}^{\left(m\right)}\right) \end{array}$$

(44) $$\begin{array}{cc} = & \sum_{m\in [2]}\sum_{l\in [2]}\left(\frac{{\bar{c}}_{m,l,1}}{\alpha_{n}-f_{m,l,1}}A_{m,l,1}B_{m,l,1}+\frac{{\bar{c}}_{m,l,2}}{\alpha_{n}-f_{m,l,2}}A_{m,l,2}B_{m,l,2}\right)+\sum_{z\in [4]}\alpha_{n}^{z-1}I_{z}, \end{array}$$

where for all z∈[4], $I_{z}={\tilde{Z}}_{1,z}+{\tilde{Z}}_{2,z}$. For all n∈[14:21], $Y_{n}$ can be written as

(45) $$\begin{array}{cc} Y_{n}= & {\bar{A}}_{n,1}^{\left(3\right)}{\bar{B}}_{n,1}^{\left(3\right)}+{\bar{A}}_{n,2}^{\left(3\right)}{\bar{B}}_{n,2}^{\left(3\right)} \end{array}$$

(46) $$\begin{array}{cc} = & \sum_{l\in [2]}\left(\frac{f_{3,l,2}-f_{3,l,1}}{\alpha_{n}-f_{3,l,1}}A_{3,l,1}B_{3,l,1}+\frac{f_{3,l,1}-f_{3,l,2}}{\alpha_{n}-f_{3,l,2}}A_{3,l,2}B_{3,l,2}\right)+\sum_{z\in [3]}\alpha_{n}^{z-1}{\bar{Z}}_{3,z} \end{array}$$

The worker node computation complexity is

(47) $$\begin{array}{c} C_{w}=O\left(\lambda \kappa \mu \right). \end{array}$$

Furthermore, under the settings of this example, the response generated by worker node *n*, n∈[21] in the baseline scheme is ${\left(\sum_{l\in [2]}{\bar{A}}_{n,l}^{\left(m\right)}{\bar{B}}_{n,l}^{\left(m\right)}\right)}_{m\in M_{n}}$, meaning the computational complexity at worker node *n* is the same as that shown in (47).

#### 4.1.3. Decoding

Let us assume that the worker node 2 is a straggler. The sink node downloads the responses $\left\{Y_{n}|n\in \left\{1\right\}\cup \left[3:20\right]\right\}$. In the following, we demonstrate that the desired matrix products ${\left(A_{m,l}B_{m,l}\right)}_{m\in [3],l\in [4]}={\left(A_{m,l,i}B_{m,l,i}\right)}_{m\in [3],l\in [2],i\in [2]}$ can be recovered from $\left\{Y_{n}|n\in \left\{1\right\}\cup \left[3:20\right]\right\}$.

For all a∈[λ],b∈[μ], we can rewrite $\left\{Y_{n}\left(a,b\right)|n\in \left\{1\right\}\cup \left[3:20\right]\right\}$ in the following matrix form:

(48) $$\begin{array}{cc} \; & {\left[Y_{1}\left(a,b\right)\;Y_{3}\left(a,b\right)\;Y_{4}\left(a,b\right)\;\cdots \;Y_{13}\left(a,b\right)\right]}^{T}\\ \; & =\left[\begin{array}{cccccccc} \frac{1}{\alpha_{1}-f_{1,1,1}} & \frac{1}{\alpha_{1}-f_{1,1,2}} & \cdots & \frac{1}{\alpha_{1}-f_{2,2,2}} & 1 & \alpha_{1} & \alpha_{1}^{2} & \alpha_{1}^{3}\\ \frac{1}{\alpha_{3}-f_{1,1,1}} & \frac{1}{\alpha_{3}-f_{1,1,2}} & \cdots & \frac{1}{\alpha_{3}-f_{2,2,2}} & 1 & \alpha_{3} & \alpha_{3}^{2} & \alpha_{3}^{3}\\ \frac{1}{\alpha_{4}-f_{1,1,1}} & \frac{1}{\alpha_{4}-f_{1,1,2}} & \cdots & \frac{1}{\alpha_{4}-f_{2,2,2}} & 1 & \alpha_{4} & \alpha_{4}^{2} & \alpha_{4}^{3}\\ \vdots & \vdots & \vdots & \vdots & \vdots & \vdots & \vdots & \vdots \\ \frac{1}{\alpha_{13}-f_{1,1,1}} & \frac{1}{\alpha_{13}-f_{1,1,2}} & \cdots & \frac{1}{\alpha_{13}-f_{2,2,2}} & 1 & \alpha_{13} & \alpha_{13}^{2} & \alpha_{13}^{3} \end{array}\right]\left[\begin{array}{c} {\bar{c}}_{1,1,1}\left(A_{1,1,1}B_{1,1,1}\right)\left(a,b\right)\\ {\bar{c}}_{1,1,2}\left(A_{1,1,2}B_{1,1,2}\right)\left(a,b\right)\\ \vdots \\ {\bar{c}}_{2,2,2}\left(A_{2,2,2}B_{2,2,2}\right)\left(a,b\right)\\ I_{1}\left(a,b\right)\\ I_{2}\left(a,b\right)\\ I_{3}\left(a,b\right)\\ I_{4}\left(a,b\right) \end{array}\right], \end{array}$$

(49) $$\begin{array}{cc} \; & {\left[Y_{14}\left(a,b\right)\;Y_{15}\left(a,b\right)\;\cdots \;Y_{20}\left(a,b\right)\right]}^{T}\\ \; & =\left[\begin{array}{ccccccc} \frac{1}{\alpha_{14}-f_{3,1,1}} & \frac{1}{\alpha_{14}-f_{3,1,2}} & \frac{1}{\alpha_{14}-f_{3,2,1}} & \frac{1}{\alpha_{14}-f_{3,2,2}} & 1 & \alpha_{14} & \alpha_{14}^{2}\\ \frac{1}{\alpha_{15}-f_{3,1,1}} & \frac{1}{\alpha_{15}-f_{3,1,2}} & \frac{1}{\alpha_{15}-f_{3,2,1}} & \frac{1}{\alpha_{15}-f_{3,2,2}} & 1 & \alpha_{15} & \alpha_{15}^{2}\\ \vdots & \vdots & \vdots & \vdots & \vdots & \vdots & \vdots \\ \frac{1}{\alpha_{20}-f_{3,1,1}} & \frac{1}{\alpha_{20}-f_{3,1,2}} & \frac{1}{\alpha_{20}-f_{3,2,1}} & \frac{1}{\alpha_{20}-f_{3,2,2}} & 1 & \alpha_{20} & \alpha_{20}^{2} \end{array}\right]\left[\begin{array}{c} \left(A_{3,1,1}B_{3,1,1}\right)\left(a,b\right)\\ \left(A_{3,1,2}B_{3,1,2}\right)\left(a,b\right)\\ \left(A_{3,2,1}B_{3,2,1}\right)\left(a,b\right)\\ \left(A_{3,2,2}B_{3,2,2}\right)\left(a,b\right)\\ {\bar{Z}}_{3,1}\left(a,b\right)\\ {\bar{Z}}_{3,2}\left(a,b\right)\\ {\bar{Z}}_{3,3}\left(a,b\right) \end{array}\right], \end{array}$$

Because the terms $\alpha_{\left[20\right]},{\left(f_{m,l,i}\right)}_{m\in [3],l\in [2],i\in [2]}$ are distinct, the square Cauchy–Vandermonde matrices on the RHS of (48) and (49) have full rank, and the desired terms $({\left(A_{1,l,i}B_{1,l,i}\left(a,b\right),A_{2,l,i}B_{2,l,i}\left(a,b\right),A_{3,l,i}B_{3,l,i}\left(a,b\right)\right)}_{l\in [2],i\in [2]}$ can be recovered by solving linear systems in (48) and (49) and removing the coefficients ${\left({\bar{c}}_{m,l,i}^{\left(k\right)}\right)}_{m\in [2],l\in [2],i\in [2]}$.

The download cost is

(50) $$\begin{array}{cc} D= & \frac{19\lambda \mu }{ML\lambda \mu }=\frac{19}{12}. \end{array}$$

The decoding complexity is at most $C_{d}=O\left(\lambda \mu \right)$. Lastly, under the settings of this example, the destination node in the baseline scheme downloads the responses ${\left(\sum_{l\in [2]}{\bar{A}}_{n,l}^{\left(m\right)}{\bar{B}}_{n,l}^{\left(m\right)}\right)}_{n\in \left\{1\right\}\cup \left[3:8\right]\cup \left[13:20\right],m\in M_{n}}$, resulting in a download cost of 21λμ/(MLλμ)=7/4. According to (41), the decoding complexity for recovering ${\left(A_{1,l,i}B_{1,l,i}\right)}_{l\in [2],i\in [2]}$ from ${\left(\sum_{l\in [2]}{\bar{A}}_{n,l}^{\left(1\right)}{\bar{B}}_{n,l}^{\left(1\right)}\right)}_{n\in [1]\cup [3:8]}$, ${\left(A_{2,l,i}B_{2,l,i}\right)}_{l\in [2],i\in [2]}$ from ${\left(\sum_{l\in [2]}{\bar{A}}_{n,l}^{\left(2\right)}{\bar{B}}_{n,l}^{\left(2\right)}\right)}_{n\in [3:8]\cup \{13\}}$, and ${\left(A_{3,l,i}B_{3,l,i}\right)}_{l\in [2],i\in [2]}$ from ${\left(\sum_{l\in [2]}{\bar{A}}_{n,l}^{\left(3\right)}{\bar{B}}_{n,l}^{\left(3\right)}\right)}_{n\in [14:20]}$ is $O\left(\lambda \mu \right)$.

### 4.2. The General Scheme

We require $\min_{k\in [K]}\rho_{\min}^{\left(k\right)}-\left|I_{k}\right|L-2X-K_{c}+1>0$. Let ${\left(\alpha_{n}\right)}_{n\in [N]},{\left(f_{m,l,i}\right)}_{m\in \left[M\right],l\in \left[\ell \right],i\in \left[K_{c}\right]}$ be N+ML distinct elements from $F_{q}$. For all $m\in \left[M\right],l\in \left[\ell \right],i\in \left[K_{c}\right]$, let us define

(51) $$\begin{array}{cc} A_{m,l,i} & =A_{m,(l-1)Kc+i} \end{array}$$

(52) $$\begin{array}{cc} B_{m,l,i} & =B_{m,(l-1)Kc+i}, \end{array}$$

where *L* matrices from either $A_{m}$ or $B_{m}$ are partitioned into *ℓ* sub-batches, each containing $K_{c}$ matrices.

#### 4.2.1. Construction of Encoding Functions

In this subsection, we present the construction of encoding functions f and g. For all $n\in \left[N\right],m\in M_{n},l\in \left[\ell \right]$, let us define the following constants:

(53) $$\begin{array}{c} \Delta_{n,m,l}=\prod_{i\in [K_{c}]}\left(\alpha_{n}-f_{m,l,i}\right). \end{array}$$

Let ${\left(Z_{m,l,x}^{\left(A\right)}\right)}_{m\in [M],l\in [\ell ],x\in [X]}$ be a total of MℓX uniformly i.i.d. random matrices over $F_{q}^{\lambda \times \kappa }$; let ${\left(Z_{m,l,x}^{\left(B\right)}\right)}_{m\in [M],l\in [\ell ],x\in [X]}$ be a total of MℓX uniformly i.i.d. random matrices over $F_{q}^{\kappa \times \mu }$.

For all $m\in \left[M\right],n\in R_{m}$, the share uploaded to worker node *n* by source node $S_{A,m}$ is constructed as follows:

(54) $$\begin{array}{cc} {\tilde{A}}_{n}^{\left(m\right)} & =\left({\bar{A}}_{n,1}^{\left(m\right)},{\bar{A}}_{n,2}^{\left(m\right)},\cdots ,{\bar{A}}_{n,\ell }^{\left(m\right)}\right), \end{array}$$

where for all l∈[ℓ],

(55) $$\begin{array}{cc} {\bar{A}}_{n,l}^{\left(m\right)} & =\Delta_{n,m,l}\left(\sum_{i\in [K_{c}]}\frac{1}{\alpha_{n}-f_{m,l,i}}A_{m,l,i}+\sum_{x\in [X]}\alpha_{n}^{x-1}Z_{m,l,x}^{\left(A\right)}\right), \end{array}$$

and the share uploaded to worker node *n* by source node $S_{B,m}$ is constructed as follows:

(56) $$\begin{array}{cc} {\tilde{B}}_{n}^{\left(m\right)} & =\left({\bar{B}}_{n,1}^{\left(m\right)},{\bar{B}}_{n,2}^{\left(m\right)},\cdots ,{\bar{B}}_{n,\ell }^{\left(m\right)}\right), \end{array}$$

where for all l∈[ℓ],

(57) $$\begin{array}{cc} {\bar{B}}_{n,l}^{\left(m\right)} & =\sum_{i\in [K_{c}]}\frac{1}{\alpha_{n}-f_{m,l,i}}B_{m,l,i}+\sum_{x\in [X]}\alpha_{n}^{x-1}Z_{m,l,x}^{\left(B\right)}, \end{array}$$

and for all m∈[M],∈[ℓ], the matrices ${\left(A_{m,l,i}\right)}_{i\in [K_{c}]}$ and ${\left(B_{m,l,i}\right)}_{i\in [K_{c}]}$ are, respectively, encoded via an MDS$\left(\rho_{m},K_{c}\right)$ code to produce $\rho_{m}$ shares. Their shares are *X*-secure due to the MDS($\rho_{m},X$)-coded random noise matrices ${\left(Z_{m,l,x}^{\left(A\right)}\right)}_{x\in [X]}$ and ${\left(Z_{m,l,x}^{\left(B\right)}\right)}_{x\in [X]}$.

The upload cost is

(58) $$\begin{array}{cc} \; & U_{A}=\frac{\sum_{m\in [M]}\rho_{m}\ell \lambda \kappa }{ML\lambda \kappa }=\frac{\sum_{m\in [M]}\rho_{m}}{MK_{c}}, \end{array}$$

(59) $$\begin{array}{cc} \; & U_{B}=\frac{\sum_{m\in [M]}\rho_{m}\ell \kappa \mu }{ML\kappa \mu }=\frac{\sum_{m\in [M]}\rho_{m}}{MK_{c}}. \end{array}$$

The encoding procedure can be considered as products of Cauchy–Vandermonde matrices and vectors. By fast algorithms [22], the encoding complexity is

(60) $$\begin{array}{cc} \; & C_{eA}=\tilde{O}\left(\frac{\lambda \kappa \sum_{m\in [M]}\ell \rho_{m}\log^{2}\rho_{m}}{ML}\right)=\tilde{O}\left(\frac{\lambda \kappa \sum_{m\in [M]}\rho_{m}\log^{2}\rho_{m}}{MK_{c}}\right) \end{array}$$

(61) $$\begin{array}{cc} \; & C_{eB}=\tilde{O}\left(\frac{\kappa \mu \sum_{m\in [M]}\ell \rho_{m}\log^{2}\rho_{m}}{ML}\right)=\tilde{O}\left(\frac{\kappa \mu \sum_{m\in [M]}\rho_{m}\log^{2}\rho_{m}}{MK_{c}}\right). \end{array}$$

#### 4.2.2. Construction of Worker Nodes Computing Functions

In this subsection, we present the construction of computing functions $h_{\left[N\right]}$. For all $k\in \left[K\right],n\in V_{k},m\in I_{k}$, let us define

(62) $$\begin{array}{c} \Gamma_{k,n,m}=\prod_{n^{\prime }\in V_{k}\setminus R_{m}}\left(\alpha_{n}-\alpha_{n^{\prime }}\right). \end{array}$$

For all $k\in \left[K\right],n\in V_{k}$, the response $Y_{n}$ sent to the sink node by worker node *n* is constructed as follows:

(63) $$\begin{array}{cc} Y_{n}= & Y_{n}=\sum_{m\in M_{n}}\Gamma_{k,n,m}\sum_{l\in [\ell ]}{\bar{A}}_{n,l}^{\left(m\right)}{\bar{B}}_{n,l}^{\left(m\right)}. \end{array}$$

Note that for all $k\in \left[K\right],n\in V_{k}$, $M_{n}=\left\{m\in \left[M\right]|n\in R_{m}\right\}=\left\{m\in I_{k}|n\in R_{m}\right\}$, thus $M_{n}\subseteq I_{k}$. For each $k\in \left[K\right],n\in V_{k}$, we obtain

(64) $$\begin{array}{cc} Y_{n}= & \sum_{m\in M_{n}}\Gamma_{k,n,m}\sum_{l\in [\ell ]}{\bar{A}}_{n,l}^{\left(m\right)}{\bar{B}}_{n,l}^{\left(m\right)}\\ = & \sum_{m\in I_{k}}\Gamma_{k,n,m}\sum_{l\in [\ell ]}(\sum_{i\in [K_{c}]}\sum_{i^{\prime }\in \left[K_{c}\right]}\frac{\Delta_{n,m,l}}{\left(\alpha_{n}-f_{m,l,i}\right)\left(\alpha_{n}-f_{m,l,i^{\prime }}\right)}A_{m,l,i}B_{m,l,i^{\prime }}\\ \; & \;+\sum_{i\in [K_{c}]}\sum_{x\in [X]}\frac{\Delta_{n,m,l}}{\alpha_{n}-f_{m,l,i}}\alpha_{n}^{x-1}A_{m,l,i}Z_{m,l,x}^{\left(B\right)}\\ \; & \;+\sum_{x\in [X]}\sum_{i\in [K_{c}]}\frac{\Delta_{n,m,l}}{\alpha_{n}-f_{m,l,i}}\alpha_{n}^{x-1}Z_{m,l,x}^{\left(A\right)}B_{m,l,i} \end{array}$$

(65) $$\begin{array}{cc} \; & \;+\sum_{x\in [X]}\sum_{t\in [X]}\Delta_{n,m,l}\alpha_{n}^{x+t-2}Z_{m,l,x}^{\left(A\right)}Z_{m,l,t}^{\left(B\right)}) \end{array}$$

(66) $$\begin{array}{cc} = & \sum_{m\in I_{k}}\Gamma_{k,n,m}\sum_{l\in [\ell ]}\left(\sum_{i\in [K_{c}]}\frac{c_{m,l,i}}{\alpha_{n}-f_{m,l,i}}A_{m,l,i}B_{m,l,i}+\sum_{z\in [2X+K_{c}-1]}\alpha_{n}^{z-1}Z_{m,l,z}\right), \end{array}$$

(67) $$\begin{array}{cc} = & \sum_{m\in I_{k}}\sum_{l\in [\ell ]}\sum_{i\in [K_{c}]}\frac{c_{m,l,i}\Gamma_{k,n,m}}{\alpha_{n}-f_{m,l,i}}A_{m,l,i}B_{m,l,i}+\sum_{m\in I_{k}}\sum_{z\in [2X+K_{c}-1]}\Gamma_{k,n,m}\alpha_{n}^{z-1}{\bar{Z}}_{m,z}, \end{array}$$

(68) $$\begin{array}{cc} = & \sum_{m\in I_{k}}\sum_{l\in [\ell ]}\sum_{i\in [K_{c}]}\frac{{\bar{c}}_{m,l,i}^{\left(k\right)}}{\alpha_{n}-f_{m,l,i}}A_{m,l,i}B_{m,l,i}+\sum_{m\in I_{k}}\sum_{z\in [|V_{k}|-\rho_{m}+2X+K_{c}-1]}\alpha_{n}^{z-1}{\tilde{Z}}_{m,z}^{\left(k\right)}, \end{array}$$

(69) $$\begin{array}{cc} = & \sum_{m\in I_{k}}\sum_{l\in [\ell ]}\sum_{i\in [K_{c}]}\frac{{\bar{c}}_{m,l,i}^{\left(k\right)}}{\alpha_{n}-f_{m,l,i}}A_{m,l,i}B_{m,l,i}+\sum_{z\in [|V_{k}|-\rho_{\min}^{\left(k\right)}+2X+K_{c}-1]}\alpha_{n}^{z-1}I_{z}^{\left(k\right)}. \end{array}$$

where (65) holds according to (55) and (57), and the fact that for all $k\in \left[K\right],n\in V_{k}$ and $m\in I_{k}\setminus M_{n}$, we obtain $R_{m}\cap \left\{n\right\}=⌀$; thus, $n\in V_{k}\setminus R_{m},\Gamma_{k,n,m}=0$. (66) is obtained by combining like terms; for all $m\in I_{k},z\in \left[2X+K_{c}-1\right],l\in \left[\ell \right]$, the undesired terms $Z_{m,l,z}$ are various linear combinations of products of matrices $A_{m,l,i},B_{m,l,i},Z_{m,l,x}^{\left(A\right)}Z_{m,l,t}^{\left(B\right)}$, whose exact forms are irrelevant. In (67), for all $m\in I_{k},z\in \left[2X+K_{c}-1\right]$, ${\bar{Z}}_{m,z}=\sum_{l\in [\ell ]}Z_{m,l,z}$. In (68), we combine like terms again. For all $m\in I_{k},z\in \left[\right|V_{k}|-\rho_{m}+2X+K_{c}-1:|V_{k}\left|-\rho_{\min}^{\left(k\right)}+2X+K_{c}-1\right]$, let us set ${\tilde{Z}}_{m,z}^{\left(k\right)}=0$. In (69), for all $z\in [|V_{k}|-\rho_{\min}^{\left(k\right)}+2X+K_{c}-1]$, $I_{z}^{\left(k\right)}=\sum_{m\in I_{k}}{\tilde{Z}}_{m,z}^{\left(k\right)}$. For all $m\in I_{k},l\in \left[\ell \right],i\in \left[K_{c}\right]$, $c_{m,l,i}=\prod_{i^{\prime }\in \left[K_{c}\right]\setminus \left\{i\right\}}\left(f_{m,l,i}-f_{m,l,i^{\prime }}\right)$ is the remainder of the polynomial division $\Delta_{n,m,l}/{\left(\alpha_{n}-f_{m,l,i}\right)}^{2}$ (with respect to $\alpha_{n}$); ${\bar{c}}_{m,l,i}^{\left(k\right)}=c_{m,l,i}\prod_{n^{\prime }\in V_{k}\setminus R_{m}}\left(f_{m,l,i}-\alpha_{n^{\prime }}\right)$, where $\prod_{n^{\prime }\in V_{k}\setminus R_{m}}\left(f_{m,l,i}-\alpha_{n^{\prime }}\right)$ is the remainder of the polynomial division $\Gamma_{k,n,m}/\left(\alpha_{n}-f_{m,l,i}\right)$ (with respect to $\alpha_{n}$).

The worker node computation complexity is

(70) $$\begin{array}{c} C_{w}=\tilde{O}\left(\frac{\sum_{n\in [N]}\sum_{m\in M_{n}}\ell \lambda \kappa \mu }{ML}\right)=O\left(\frac{\lambda \kappa \mu \sum_{m\in [M]}\rho_{m}}{MK_{c}}\right). \end{array}$$

**Remark** **5.** *By the definition of connected components in hypergraphs, every hyperedge $R_{⇕}\in E_{k}$, $m\in I_{k}$ satisfies $R_{⇕}\subseteq V_{k}$, and for any vertex $v\in V_{k}$, all hyperedges containing v belong exclusively to $E_{k}$. This structural property has a direct implication for the LESDBMM problem: for each k∈[K], the matrices $A_{m}$ and $B_{m}$, $m\in I_{k}$ are shared only among the worker nodes in $V_{k}$, and conversely, each worker node in $V_{k}$ receives shares exclusively from matrices ${\left(A_{m},B_{m}\right)}_{m\in I_{k}}$. Consequently, the recovery of the matrix products ${\left(A_{m,l}B_{m,l}\right)}_{m\in I_{k},l\in \left[L\right]}$ can be accomplished solely using the responses from the worker nodes in $V_{k}$, together with computing functions $h_{V_{k}}$ and the corresponding decoding strategy. Importantly, this recovery process is independent of the workers outside $V_{k}$ as well as the decoding strategies employed in other connected components.*

**Remark** **6.** *For any k∈[K], although only the worker nodes in $R_{m}$ hold shares of the matrices $A_{m}$ and $B_{m}$, the use of the CSA null shaper ($\Gamma_{k,n,m}$) ensures that every worker node n in $V_{k}$ possesses the terms ${\left(\Gamma_{k,n,m}\sum_{l\in [\ell ]}{\bar{A}}_{n,l}^{\left(m\right)}{\bar{B}}_{n,l}^{\left(m\right)}\right)}_{m\in I_{k}}$, as illustrated in (65). Moreover, the CSA null shaper preserves the structure of CSA encoding: the desired symbols are aligned with the Cauchy terms, while the undesired interference terms are aligned with the Vandermonde terms, as shown in (68). Consequently, the problem of decoding the matrix products ${\left(A_{m,l}B_{m,l}\right)}_{m\in I_{k},l\in \left[L\right]}$ reduces to the problem of CSA decoding without encoding locality in the presence of stragglers [8].*

#### 4.2.3. Construction of Decoding Functions

In this subsection, we present the construction of decoding functions ${\left(d_{T}\right)}_{T\subset [N]}$. For all k∈[K], let us define $T_{k}=V_{k}\cap T$, representing the non-stragglers among worker nodes $V_{k}$; note that $|T_{k}\left|\geq \right|V_{k}|-S\geq \gamma_{k}$. The sink node downloads the responses ${\left(Y_{T_{k}\left(i\right)}\right)}_{k\in \left[K\right],i\in \left[\gamma_{k}\right]}$. For all k∈[K], let us define

(71) $$\begin{array}{cc} C_{k}= & \left[\begin{array}{cccc} \frac{1}{\alpha_{T_{k}\left(1\right)}-f_{I_{k}\left(1\right),1,1}} & \frac{1}{\alpha_{T_{k}\left(1\right)}-f_{I_{k}\left(1\right),1,2}} & \cdots & \frac{1}{\alpha_{T_{k}\left(1\right)}-f_{I_{k}\left(\right|I_{k}\left|\right),\ell ,K_{c}}}\\ \frac{1}{\alpha_{T_{k}\left(2\right)}-f_{I_{k}\left(1\right),1,1}} & \frac{1}{\alpha_{T_{k}\left(2\right)}-f_{I_{k}\left(1\right),1,2}} & \cdots & \frac{1}{\alpha_{T_{k}\left(2\right)}-f_{I_{k}\left(\right|I_{k}\left|\right),\ell ,K_{c}}}\\ \vdots & \vdots & \vdots & \vdots \\ \frac{1}{\alpha_{T_{k}\left(\gamma_{k}\right)}-f_{I_{k}\left(1\right),1,1}} & \frac{1}{\alpha_{T_{k}\left(\gamma_{k}\right)}-f_{I_{k}\left(1\right),1,2}} & \cdots & \frac{1}{\alpha_{T_{k}\left(\gamma_{k}\right)}-f_{I_{k}\left(\right|I_{k}\left|\right),\ell ,K_{c}}} \end{array}\right], \end{array}$$

(72) $$\begin{array}{cc} V_{k}= & \left[\begin{array}{cccc} 1 & \alpha_{T_{k}\left(1\right)} & \cdots & \alpha_{T_{k}\left(1\right)}^{|V_{k}|-\rho_{\min}^{\left(k\right)}+2X+K_{c}-2}\\ 1 & \alpha_{T_{k}\left(2\right)} & \cdots & \alpha_{T_{k}\left(2\right)}^{|V_{k}|-\rho_{\min}^{\left(k\right)}+2X+K_{c}-2}\\ \vdots & \vdots & \vdots & \vdots \\ 1 & \alpha_{T_{k}\left(\gamma_{k}\right)} & \cdots & \alpha_{T_{k}\left(\gamma_{k}\right)}^{|V_{k}|-\rho_{\min}^{\left(k\right)}+2X+K_{c}-2} \end{array}\right]. \end{array}$$

For all k∈[K],a∈[λ],b∈[μ], we can rewrite the downloaded symbols $\left\{Y_{T_{k}\left(i\right)}\left(a,b\right)|i\in \left[\gamma_{k}\right]\right\}$ in the following matrix form:

(73) $$\begin{array}{cc} \left[\begin{array}{c} Y_{T_{k}\left(1\right)}\left(a,b\right)\\ Y_{T_{k}\left(2\right)}\left(a,b\right)\\ \cdots \\ Y_{T_{k}\left(\gamma_{k}\right)}\left(a,b\right) \end{array}\right]\; & =\left[C_{k}\;V_{k}\right]\left[\begin{array}{c} {\bar{c}}_{I_{k}\left(1\right),1,1}\left(A_{I_{k}\left(1\right),1,1}B_{I_{k}\left(1\right),1,1}\right)\left(a,b\right)\\ {\bar{c}}_{I_{k}\left(1\right),1,2}\left(A_{I_{k}\left(1\right),1,2}B_{I_{k}\left(1\right),1,2}\right)\left(a,b\right)\\ \vdots \\ {\bar{c}}_{I_{k}\left(\right|I_{k}\left|\right),\ell ,K_{c}}\left(A_{I_{k}\left(\right|I_{k}\left|\right),\ell ,K_{c}}B_{I_{k}\left(\right|I_{k}\left|\right),\ell ,K_{c}}\right)\left(a,b\right)\\ I_{1}^{\left(k\right)}\left(a,b\right)\\ I_{2}^{\left(k\right)}\left(a,b\right)\\ \vdots \\ I_{|V_{k}|-\rho_{\min}^{\left(k\right)}+2X+K_{c}-1}^{\left(k\right)}\left(a,b\right) \end{array}\right] \end{array}$$

Because the terms $\alpha_{V_{k}},{\left(f_{m,l,i}\right)}_{m\in I_{k},l\in \left[\ell \right],i\in \left[K_{c}\right]}$ are distinct, the square Cauchy–Vandermonde matrix on the RHS of (73) has full rank, and the desired terms ${\left(\left(A_{m,l,i}B_{m,l,i}\right)\left(a,b\right)\right)}_{m\in I_{k},l\in \left[\ell \right],i\in \left[K_{c}\right]}$ can be recovered by solving linear systems in (73) and removing the coefficients ${\left({\bar{c}}_{m,l,i}^{\left(k\right)}\right)}_{m\in I_{k},l\in \left[\ell \right],i\in \left[K_{c}\right]}$. This completes the proof of the correctness of the decoding functions. The download cost is

(74) $$\begin{array}{cc} D= & \frac{\sum_{k\in \left[K\right],i\in \left[\gamma_{k}\right]}\left|Y_{T_{k}\left(i\right)}\right|}{ML\lambda \mu }=\frac{\sum_{k\in [K]}\gamma_{k}}{ML}. \end{array}$$

By fast algorithms [23], the complexity of solving a linear system defined by a n×n Cauchy- Vandermonde matrix is $\tilde{O}\left(n\log^{2}n\right)$; thus, the decoding complexity is at most

(75) $$\begin{array}{c} C_{d}=\tilde{O}\left(\frac{\lambda \mu \sum_{k\in [K]}\gamma_{k}\log^{2}\gamma_{k}}{ML}\right). \end{array}$$

## 5. Concluding Remarks

This work proposes the first LESDBMM scheme based on the idea of CSA codes and CSA null shaper, which can be viewed as a generalization of CSA codes for CDBMM [8] to the LESDBMM setting. The proposed scheme can be further extended in two promising directions. First, following the approach of [8], which combines CSA codes for CDBMM with EP codes to obtain GCSA codes, one can generalize our construction to support matrix partitioning while preserving batch processing capabilities. Second, by allowing the worker nodes to share common randomness that is independent of the input matrices (as in [18]), the scheme can be enhanced to guarantee information-theoretic privacy: the sink node learns nothing about the input matrices beyond the desired matrix products. In addition, the current design focuses on the worst-case scenario, assuming that the number of stragglers reaches the maximum tolerable threshold. However, in practice, the number of stragglers is unpredictable; it is worthwhile to explore how to fully leverage all available worker nodes to further improve both computation and communication efficiency.

## Figures and Tables

**Table 1:** Performance comparison of a baseline scheme and our scheme. The baseline scheme independently encodes $A_{m}$ and $B_{m}$, computes, and decodes ${\left(A_{m,l}B_{m,l}\right)}_{l\in [L]}$ for each pair of source nodes $\left(S_{A,m},S_{B,m}\right)$ using CSA codes [8] in a straightforward manner. $R=L+2X+K_{c}-1$, P ranges over all subsets of T such that $|P\cap R_{m}|\geq R$ for all m∈[M].

	Our Scheme	Baseline Scheme
Straggler Threshold	$\min_{k\in [K]}\rho_{\min}^{\left(k\right)}-\left|I_{k}\right|L-2X-K_{c}+1$	$\min_{m\in [M]}\rho_{m}-L-2X-K_{c}+1$
Upload Cost	$\left(\frac{\sum_{m\in [M]}\rho_{m}}{MK_{c}},\frac{\sum_{m\in [M]}\rho_{m}}{MK_{c}}\right)$	$\left(\frac{\sum_{m\in [M]}\rho_{m}}{MK_{c}},\frac{\sum_{m\in [M]}\rho_{m}}{MK_{c}}\right)$
Dowload Cost	$\frac{\sum_{k\in [K]}\gamma_{k}}{ML}$	$\frac{\min_{P}\sum_{n\in P}\left|M_{n}\right|}{ML}$
Encoding Complexity	$(\tilde{O}\left(\frac{\lambda \kappa \sum_{m\in [M]}\rho_{m}\log^{2}\rho_{m}}{MK_{c}}\right),$ $\tilde{O}\left(\frac{\kappa \mu \sum_{m\in [M]}\rho_{m}\log^{2}\rho_{m}}{MK_{c}}\right))$	$(\tilde{O}\left(\frac{\lambda \kappa \sum_{m\in [M]}\rho_{m}\log^{2}\rho_{m}}{MK_{c}}\right),$ $\tilde{O}\left(\frac{\kappa \mu \sum_{m\in [M]}\rho_{m}\log^{2}\rho_{m}}{MK_{c}}\right))$
Worker Nodes Computation Complexity	$O\left(\frac{\lambda \kappa \mu \sum_{m\in [M]}\rho_{m}}{MK_{c}}\right)$	$O\left(\frac{\lambda \kappa \mu \sum_{m\in [M]}\rho_{m}}{MK_{c}}\right)$
Decoding Complexity	$\tilde{O}\left(\frac{\lambda \mu \sum_{k\in [K]}\gamma_{k}\log^{2}\gamma_{k}}{ML}\right)$	$\tilde{O}\left(\frac{\lambda \mu R\log^{2}R}{L}\right)$

## Data Availability

The data are contained within the article.

## Abbreviations

The following abbreviations are used in this manuscript:

LESDBMM	Locally Encoded Secure Distributed Batch Matrix Multiplication
CSA	Cross-Subspace Alignment
GCSA-NA	Generalized Cross-Subspace Alignment with Noise Alignemnt
CDBMM	Coded Distributed Batch Matrix Multiplication
MDS	Maximum Distrance Separable
EP	Entangled Polynomial
LCC	Lagrange Coded Computing
GCSA	Generalized Cross-Subspace Alignment
SDBMM	Secure Distributed Batch Matrix Multiplication
GXSTPLC	X-Secure T-Private Linear Computation Based on Graph-Based Replicated Storage
GRS	Generalized Reed–Solomon
